# Radiologically Defined Sarcopenia as a Biomarker for Frailty and Malnutrition in Head and Neck Skin Cancer Patients

**DOI:** 10.3390/jcm12103445

**Published:** 2023-05-13

**Authors:** Aniek T. Zwart, Laurence M. C. Kok, Julius de Vries, Marloes S. van Kester, Rudi A. J. O. Dierckx, Geertruida H. de Bock, Anouk van der Hoorn, Gyorgy B. Halmos

**Affiliations:** 1 Department of Epidemiology, University Medical Center Groningen, University of Groningen, 9713 GZ Groningen, The Netherlands; g.h.de.bock@umcg.nl; 2 Department of Radiology, University Medical Center Groningen, University of Groningen, 9713 GZ Groningen, The Netherlands; 3 Department of Otolaryngology and Head and Neck Surgery, University Medical Center Groningen, University of Groningen, 9713 GZ Groningen, The Netherlands; 4 Department of Dermatology, University Medical Center Groningen, University of Groningen, 9713 GZ Groningen, The Netherlands; 5 Department of Dermatology, Haga Hospital Location Leyweg (Hagaziekenhuis), 2545 AA The Hague, The Netherlands

**Keywords:** head and neck neoplasms, skin neoplasms, postoperative complications, geriatric assessment, frail elderly, sarcopenia

## Abstract

The aim of this study was to evaluate whether radiologically defined sarcopenia, or a low skeletal muscle index (SMI), could be used as a practical biomarker for frailty and postoperative complications (POC) in patients with head and neck skin cancer (HNSC). This was a retrospective study on prospectively collected data. The L3 SMI (cm^2^/m^2^) was calculated with use of baseline CT or MRI neck scans and low SMIs were defined using sex-specific cut-off values. A geriatric assessment with a broad range of validated tools was performed at baseline. POC was graded with the Clavien–Dindo Classification (with a grade of > II as the cut-off). Univariate and multivariable regression analyses were performed with low SMIs and POC as the endpoints. The patients’ (n = 57) mean age was 77.0 ± 9 years, 68.4% were male, and 50.9% had stage III–IV cancer. Frailty was determined according to Geriatric 8 (G8) score (OR 7.68, 95% CI 1.19–49.66, *p* = 0.032) and the risk of malnutrition was determined according to the Malnutrition Universal Screening Tool (OR 9.55, 95% CI 1.19–76.94, *p* = 0.034), and these were independently related to low SMIs. Frailty based on G8 score (OR 5.42, 95% CI 1.25–23.49, *p* = 0.024) was the only variable related to POC. However, POC was more prevalent in patients with low SMIs (∆ 19%, OR 1.8, 95% CI 0.5–6.0, *p* = 0.356).To conclude, a low SMI is a practical biomarker for frailty and malnutrition in HNSC. Future research should be focused on interventions based on low SMI scores and assess the effect of the intervention on SMI, frailty, malnutrition, and POC.

## 1. Introduction

Older patients have a higher chance of developing HNSC due to the cumulative damages from solar UV radiation, and this is a population that is expanding as our society ages [1,2,3,4]. Surgery is the primary treatment choice for HNSC; however, primary radiotherapy can be an alternative to surgery in selected cases. In general, surgical interventions for HNSC are relatively simple with local excision, but extensive surgery can be necessary for cases of advanced disease. Preoperative screening for this population is essential as older patients may have more comorbidities, functional impairments, psychological issues, and poorer social support, all of which can affect perioperative risk [5]. Hence, a multidisciplinary approach and personalized treatment are important for decision-making [6,7].

The Comprehensive Geriatric Assessment (CGA) is a multidimensional and interdisciplinary assessment and is the gold standard for identifying frail patients [6,8]. However, the CGA is time-intensive, partially subjective, requires the active participation of the patient, and can be strenuous for the patient or clinician. Therefore, shorter frailty screening questionnaires, such as the Geriatric 8 (G8) and the Groningen Frailty Indicator (GFI) are also available. Screening for frailty with the G8 is promising as it is related to postoperative complications (POC) [9], guideline deviations [10], and declined quality of life in HNSC patients [11]. Although shorter, these frailty screening tools still require the active participation of the patient, and the frailest patients tend to not return questionnaires [12]. A simple, objective method to assess frailty and the risk of POC could be helpful to overcome these problems.

SMI is considered a surrogate biomarker for total body skeletal muscle mass [13] and could be a fast, objective biomarker for frailty and POC in HNSC patients. Generally, neck imaging for HNSC is reserved for more complex or advanced cases. SMI can reliably be measured on CT and MRI neck scans that are conducted during oncological work-up [14,15], and it provides a convenient, objective, and less time-intensive tool relative to the CGA. A low SMI, also referred to as radiologically defined sarcopenia, has already emerged as a predictor for adverse clinical outcomes, including POC and frailty in patients with mucosal head and neck cancers (mHNC) [16,17,18]. The impact of a low SMI in HNSC could be considerable as a recent meta-analysis found that low SMIs were related to lower progression-free survival and lower overall survival in patients diagnosed with malignant cutaneous melanoma who had been treated with palliative immunotherapy [19].

However, the clinical value of SMI for predicting frailty and POC is unknown in HNSC, and insights could be beneficial for multidisciplinary teams when making treatment decisions or selecting patients for pre-habilitation, particularly in an older population. Therefore, in the present study, the aims were to: (1) determine SMI using baseline CT or MRI neck scans conducted during oncological work-up, (2) analyze the relationship between frailty and (low) SMI, and (3) investigate the impact of (low) SMI and frailty on the occurrence of POC in patients with HNSC.

## 2. Materials and Methods

Patients in this retrospective cohort study were prospectively enrolled in the Oncological Life Study (OncoLifeS) databiobank [20] after obtaining written informed consent. This large-scale, institutional oncological databiobank collects and stores the following details of adult patients diagnosed with cancer: clinical and treatment data, comorbidities, lifestyle, radiological and pathological findings, biomaterials, quality of life, and long-term outcomes. The OncoLifeS databiobank has been approved by the Medical Ethical Committee of the University Medical Center Groningen (UMCG) and is registered in the Dutch Trial Register under the registration number NL7839. The scientific board of OncoLifeS gave its permission for this study.

### 2.1. Patient Population and Data Collection

Between October 2014 and October 2018, 197 patients with HNSC were included in OncoLifeS. The patients were treated according to national guidelines within the multidisciplinary head and neck tumor board and, if applicable, the melanoma board. Eligibility criteria for the present study were patients who had been surgically treated for HNSC in the UMCG with follow-up data on POC, sufficient neck imaging at baseline, and a geriatric assessment at baseline (n = 65). Patients without imaging data at a level of C3 (n = 5), those with too small field of view (n = 2), or those with too much angulation in the cervical spine (n = 1) were excluded. In total, 57 patients (28.9% of the initial sample size) were included in this study.

The baseline patient, tumor, and treatment characteristics were extracted from the OncoLifeS databiobank, including age (years), sex, body mass index (BMI, kg/m^2^), smoking status (never vs. former or current), alcohol usage (none or mild vs. heavy, as defined by the usage of two alcohol units or more per day), reason for referral (primary vs. residual or recurrent), primary tumor location, stage of disease (stage I–II vs. II–IV), tumor size (cm), tumor type, treatment intensity (minor vs. major, as defined by a surgery of > 120 min), type of anesthesia (local vs. general), and reconstructive surgery (yes vs. no). The seventh edition of the Union for International Cancer Control TNM Classification was used for defining tumor stage.

### 2.2. Frailty Screening and Geriatric Assessment

The included patients underwent a geriatric assessment on the first day of consultation using a range of validated tools (Table S1), and the outcomes were registered in OncoLifeS. The Geriatric 8 (G8) and Groningen Frailty Indicator (GFI) were used for frailty screening.

### 2.3. Quantification of Skeletal Muscle Mass

All scans were made for clinical purposes and performed using modern CT (n = 43) or MRI (1.5 Tesla, n = 9; 3 Tesla, n = 5) scanners. Most CT scans were performed with an intravenous iodine contrast (n = 42) and with the use of a soft tissue kernel of between 20 and 40 (n = 37). The CT slice thicknesses were 0.6–1.25 mm. Most MRI scans had a slice thickness of 3.0 mm without the use of an intravenous contrast (n = 13). Measurements on the MRI scans were completed on a T2, and if a T2 was not available, they were completed on a T1 (n = 4).

The SMIs were measured with CT and MRI neck scans using previously validated procedures [14,15]. In short, the third cervical vertebra (C3) was identified and the cross-sectional area (CSA, cm^2^) of the neck musculature was measured [14]. The CSA at the C3 level was converted to the CSA at the third lumbar vertebra (L3) to calculate the SMI (cm^2^/m^2^) (see Equations 1 and 2) [13,14]. A low SMI was defined using sex-specific SMI cut-off values, with an SMI of < 42.4 cm^2^/m^2^ for males and an SMI of < 30.6 cm^2^/m^2^ for females [21]. One observer (LMC) took all of the measurements and was blinded for the baseline characteristics and clinical outcomes. Before making the CSA measurements in the dataset of the present study, the performance of this observer was tested in a separate training set (with the CT n = 25 and the MRI n = 25). In addition to the main observer, the observers for the inter-observer analyses included a PhD student (ATZ) with 5 years of experience doing these measurements, a board-certified radiologist, and three medical students. All CSA measurements taken by the main observer in the dataset of the present study were visually verified by ATZ. The equations used for the calculations were:

(1) $${CSAatL3(\mathrm{cm}}^{2})=27.304+1.363\;*\;CSAatC3\left({\mathrm{cm}}^{2}\right)+0.640\;*\;Weight\left(\mathrm{kg}\right)\;+26.442\;*\;Gender\left(Gender=1\;forFemale,2\;forFemale\right)-0.671\;*\;Age\left(\mathrm{years}\right)$$

The lumbar SMI was then calculated using the formula published by Prado et al. see Formula (2) [5]

(2) $$LumbarSMI({\mathrm{cm}}^{2}/m^{2})=CMSAatL3/(height\;*\;height).$$

### 2.4. Postoperative Outcomes

POC was classified using the Clavien–Dindo Classification (CDC) with a grade of > II as a cut-off [22]. Unplanned readmission for any cause and duration of hospitalization (days) within thirty days post-surgery were recorded.

### 2.5. Statistical Analysis

Baseline characteristics, adverse postoperative outcomes, and frailty status were presented as means (standard deviations), medians (ranges), or values (%). Normality was analyzed in continuous data with a Kolmogorov–Smirnov analysis and Q–Q plots. Inter-rater observer reliability was analyzed with the Intraclass Correlation Coefficient (ICC). For the second research aim, the relationship between frailty and skeletal muscle mass was assessed by univariate and multivariable linear (with SMI being dependent) and logistic (with a low SMI being dependent) regression analyses. For the third research aim, the relationship between skeletal muscle mass, frailty, and POC was analyzed with univariate and multivariable logistic regression analyses (with a CDC grade of >II being dependent), and skeletal muscle mass, frailty, and the other baseline variables were the covariates. Statistically significant and clinically relevant variables (α < 0.05, two-sided) from the univariate regression analyses with high impacts on the dependent variable, without multicollinearity (variance inflation factors of < 3), were selected for the multivariable regression analysis. To reduce overfitting, a multivariable model with only three covariates was built. Odds ratios (ORs) or beta (B) and 95% confidence intervals (CIs) were provided. SPSS version 28 (IBM, Armonk, NY, USA) was used for the analyses.

## 3. Results

### 3.1. General Patient Characteristics

In total, 57 patients with HNSC having neck imaging and a geriatric assessment at baseline were included in the present study. Table 1 shows the patients’ baseline characteristics. The mean (SD) age of the study population was 77.1 (± 9.0) years, and a majority of the patients were male (68.4%) and had stage III–IV disease classifications (50.9%). The tumors were mostly keratinocyte carcinoma (squamous and basal cell carcinoma) (73.7%) and located on the ears (36.8%). The prevalence levels of frailty were 20.0% and 41.9% for the GFI and the G8, respectively (Table 2).

### 3.2. Predictors for Skeletal Muscle Mass

**Figure 1:**
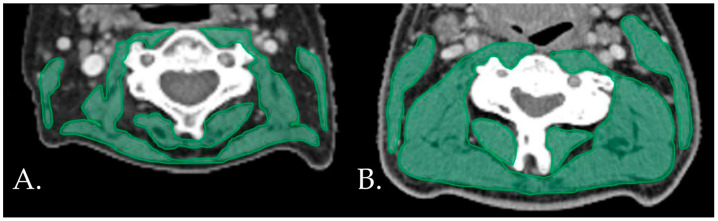
Examples of patients with (**A**) and without (**B**) low SMIs on the neck CT.s CT: computed tomography. SMI: skeletal muscle index.

The inter-rater reliability of the main observer was excellent for both the CT (ICC = 0.994, 95% CI 0.982–0.998, *p* < 0.001) and the MRI (ICC = 0.985, 95% CI 0.970–0.993, *p* < 0.001). The mean (SD) SMIs were 42.40 ± 6.75, 44.95 ± 6.01, and 26.87 ± 6.01 cm^2^/m^2^ for the total population, male patients, and female patients, respectively. Seventeen (29.8%) patients were diagnosed has having low SMIs. Figure 1 shows examples of patients with and without low SMIs. Frequencies, means, and medians for the clinical characteristics, frailty domains, and postoperative outcomes for the total population and for the patients with and without low SMIs are displayed in Table 1, Table 2 and Table 3, respectively.

The outcomes of the univariate regression analyses for low SMIs and SMIs are shown in Table 4 and Table S2. Adjusted for the type of anesthesia, the multivariable logistic regression identified frailty based on the G8 frailty screening tool scores (OR 7.68, 95% CI 1.19–49.66, *p* = 0.032), and medium-high malnutrition risk was determined according to the MUST (OR 9.55, 95% CI 1.19–76.94, *p* = 0.034) as significant variables associated with low SMIs (Table 5). After correction of alcohol usage, female sex (B −7.36, 95% CI −10.56–−4.16, *p* < 0.001) and (ex-) smokers (B 3.15, 95% CI 0.17–6.34, *p* = 0.039) remained significantly related to SMI according to the linear multivariable regression analysis (Table S3). 

### 3.3. Predictors for Postoperative Outcomes

Of all patients, 61.4% endured POCs (CDC > II) (Table 3). The univariate logistic regression with POC as the dependent variable (Table 4) showed that SMI as a continuous variable did not have a high or significant impact on POC (OR 1.02, 95% CI 0.94–1.10, *p* = 0.703). Although the occurrence of POC was more often seen in patients with low SMIs (70.6%) compared to patients with normal SMIs (51.5%), the association was not significant (OR 1.77 95% CI 0.53–5.99, *p* = 0.356). POCs did not occur in patients with local anesthesia. To generate an OR for anesthesia type, the occurrence of POC was randomly added to one patient with local anesthesia. Although not significant, general anesthesia may have had a high impact on POC (OR 8.24 95% CI 0.85–79.44, *p* = 0.068). The G8 frailty screening tool score (OR 5.42, 95% CI 1.25–23.49, *p* = 0.024) was the only variable significantly related to POC according to the univariate logistic regression analysis, and a multivariable regression analysis was therefore not conducted. Secondary outcomes showed that unplanned readmission and duration of hospitalization were equally distributed between patients with and without low SMIs.

## 4. Discussion

To our knowledge, this is the first study that quantified skeletal muscle mass with SMI in HNSC patients using CT or MRI neck scans taken during oncological work-up and assessed its clinical value. The key findings were that malnutrition risk (MUST) and frailty (G8) were independently and significantly related to radiologically defined sarcopenia (low SMI), and further, frailty (G8) was the only variable significantly related to POC. Although the difference was not significant, patients with low SMIs more often had POCs compared to patients with normal SMIs. These key findings give new insights into the interrelation of low SMIs, frailty, and POCs in patients diagnosed with HNSC.

### 4.1. Frailty, Malnutrition, and Skeletal Muscle Mass

The results of the present study are in line with other studies on frailty and low SMIs in mHNC [18,23,24,25]. Frailty and sarcopenia are not the same, and frailty is considered a geriatric syndrome while sarcopenia a disease [26]. Both are, however, related to multiple adverse clinical outcomes [27,28], and they have been found to be related to each other [18]. In this present study, a low SMI was found to be related to G8 score and not GFI score. This discrepancy in outcomes can be explained by the content of the frailty indicators. Compared to the GFI, the G8 is more focused on weight loss, BMI, mobility, and food intake, and it leans more toward a physical definition of frailty, which has a tendency to overlap more with sarcopenia [26,29]. In mHNC, previous studies have also found a significant relationship between a low SMI (with or without low muscle strength) and G8 score [23] but not with GFI score [23]. Moreover, G8 score was found to be the most suitable frailty screening tool in older adults with skin cancer [30], highlighting the importance of the found relationship between a low SMI and G8 score in this study. Officially, sarcopenia is defined as low muscle performance/strength and low muscle mass [26]. Moreover, the specificity of the G8 has been debated, and Pottel et al. and Hamaker et al. concluded that the G8 frailty screening tool is very sensitive—but not very specific—in contrast to the CGA [6,7]. Meerkerk et al. further investigated the association between frailty as measured with a geriatric assessment and a low SMI with and without low muscle strength in mHNC [23]. They found that a low SMI (without consideration of low muscle strength) was related to frailty [23]. This implies that adding muscle strength into the sarcopenia diagnosis is not beneficial for identifying frail patients, but it should be investigated if this is also the case in HNSC. Patients with low SMIs had higher risks of malnutrition in this study, which was in line with other studies [18,31]. Moreover, low SMIs could be irreversible as studies have shown that nutritional and/or exercise interventions are feasible and able to improve skeletal muscle mass in patients with mHNC [32,33], which, in turn, may improve (nutritional) health outcomes and frailty status.

### 4.2. Frailty and Postoperative Complications

De Vries et al. also analyzed the value of geriatric assessment and frailty indicators for predicting postoperative outcomes in patients diagnosed with HNSC undergoing surgery, and they found the G8 frailty indicator to be related to POC [9], which was in line with the outcome of the present study. Despite an overlapping patient cohort between the present study and the study by de Vries et al., differences were apparent regarding the definitions for POC (CDC grade of > II vs. grade of > III) and stage of disease (50.9% vs. 25.9% stage III–IV cancers). Therefore, it could be concluded that the G8 is able to predict postoperative complications in different cohorts of heterogenic HNSC patients. Moreover, the G8 has been shown to be related to other adverse health outcomes in HNSC patients, including guideline deviation [10] and declined quality of life [11]. A recent study by Valdatta et al. also observed a significant association between frailty (measured with FRAIL scores) and surgical complications in elderly patients diagnosed with non-melanoma skin cancer [34]. Therefore, screening for frailty appears to have a predictive value for adverse postoperative outcomes in skin cancer patients and should be recommended before initiating major surgery.

### 4.3. Skeletal Muscle Mass and Postoperative Complications

In mHNC patients, pre-treatment diagnosed sarcopenia has already been associated with negative clinical outcomes [17,18,27,35,36]. In this cohort, the patients with low SMIs more often developed POCs, and therefore, it appears to be a promising predictor. However, the difference was not significant, which was very likely due to the small sample size and the fact that less general anesthesia was used in sarcopenic patients, which, in turn, possibly had a high impact on POC. Low SMIs appeared to have a greater impact than SMI as a continuous variable on POC in this cohort. Sabel et al. found in their cohort of stage III melanoma patients that skeletal muscle mass qualified with decreased psoas muscle density on CT, which was independently associated with decreased disease-free survival, distant disease-free survival, and higher rates of surgical complications [37]. Measuring muscle density or adding low muscle strength to a sarcopenia diagnosis may further improve the association between skeletal muscle and POC. However, muscle density analysis using CT images was not feasible as most CT scans in the present study were generated with an intravenous iodine contrast, which is known to affect the muscle density measurements [38], and thus, no data on muscle strength were available.

### 4.4. Limitations

First, our sample size was relatively small and heterogenic in terms of tumor characteristics with a high percentage of complex cases. Therefore, caution should be made when extrapolating our findings to patients diagnosed with less complex and low-risk HNSC. Heterogeneous image techniques could be regarded as another possible limitation; however, recent research has found that CT and MRI neck imaging could be used interchangeably for skeletal muscle analysis [15]. In the present study, low muscle strength was not a criterion for sarcopenia. This could be seen as a limitation; however, a low SMI without consideration of muscle strength has been found to be associated with inferior health outcomes [39]. Moreover, others have encouraged the use of SMI and not muscle strength at the core of nutritional management strategies as skeletal muscle mass is an important metabolically active and homeostatic indicator [40]. Nevertheless, low muscle strength as an additional criterion for sarcopenia may be beneficial in HNSC cases to predict clinical outcomes. Ideally, SMI cut-off values as generated in an HNSC population should be applied to define low SMIs.

### 4.5. Strengths

First, the association between frailty and sarcopenia was assessed and their impact on postoperative outcomes was analyzed, which is highly clinically relevant. Second, patients were included prospectively and were assessed with a broad range of validated geriatric assessments and screening tools at baseline. Third, high observer reliability scores were achieved, and the observer was furthermore blinded from the clinical outcome, preventing bias. Fourth, we evaluated skeletal muscle mass using both SMIs and low SMIs to examine if certain relationships existed with or without using an SMI cut-off value.

### 4.6. Future Research

Identifying patients with low SMIs and assessing their prognostic value is fairly new in dermato-oncology, which creates many opportunities. It would be interesting to see if the prognostic value of SMI on POC can be improved. For instance, a low SMI defined using HNSC-specific SMI cut-off values may better predict postoperative outcomes than a low SMI based on mHNC SMI cut-off values. Moreover, the effect of low muscle strength on POC should be assessed. Therefore, after optimizing SMI cut-off values in HNSC, the present study should be repeated at a large-scale multicenter study to analyze the relationship between frailty, a low SMI (with and without consideration of low muscle strength), and POC in HNSC. Ideally, a multivariable regression analysis on (major) POC should be performed, including relevant clinical variables related to POC. Additionally, randomized controlled trials with interventions on low SMIs (with or without consideration of low muscle strength) should be performed to assess the effect on frailty and POC.

## 5. Conclusions

Preoperative frailty screening of elderly patients at risk for POCs is highly recommended, but it is time-intensive and could be strenuous for the patients. The present study found that malnutrition risk and frailty were independently related to low SMIs (also radiologically defined sarcopenia). Frailty, not SMI, was related to POC. Although the difference was not significant, patients with low SMIs more often had had POCs compared to patients with normal SMIs. These outcomes implied that patients with low SMIs may benefit from interventions to improve their frailty and nutritional status, which, in turn, may result in fewer complications. Therefore, identifying patients with low SMIs at baseline may help multidisciplinary teams to make treatment decisions or select patients for pre-habilitation. Hence, a low SMI is a practical and objective radiological biomarker for screening for frailty and malnutrition. However, further research is needed to assess the capability of SMIs to predict postoperative outcomes. Preoperative screening for frailty should be advised for major surgeries as frailty was the only variable significantly related to POC in this cohort of HNSC patients.

## Figures and Tables

**Table 1:** Demographic and clinical characteristics of patients surgically treated for cutaneous malignancies of the head and neck area. The data are stratified for sarcopenia diagnosis. Disease stage was defined using the seventh edition of the Union for International Cancer Control TNM Classification. * indicates other malignancies, including angiosarcoma (*n* = 2), pleomorphic dermal sarcoma (*n* = 1), and dermatofibrosarcoma protuberans (*n* = 1). ** indicates instances defined as a surgery of > 120 min. *** indicates intraoperative reconstruction or subsequent reconstructive surgery. Due to missingness, not all numbers sum up to 57. BMI = body mass index and SD = standard deviation.

	Total *n =* 57	Normal SMI *n =* 40 (70.2%)	Low SMI *n =* 17 (29.8%)
**Patient characteristics**			
Age, mean ± SD, year	77.1 ± 9.0	75.5 ± 9.0	80.9 ± 7.9
Sex			
Male	39 (68.4%)	16 (40.0%)	2 (11.8%)
Female	18 (31.6%)	24 (60.0%)	15 (88.2%)
BMI, mean ± SD, kg/cm^2^	26.9 ± 4.1	28.3 ± 3.9	23.6 ± 2.2
Smoking status			
Never	16 (34.0%)	11 (31.4%)	5 (41.7%)
Former or current	31 (66.0%)	24 (68.6%)	7 (58.3%)
Alcohol usage			
None or mild	37 (88.1%)	26 (86.7%)	11 (91.7%)
Heavy (>2 units/day)	5 (11.9%)	4 (13.3%)	1 (8.3%)
**Tumor characteristics**			
Reason for referral			
Primary	26 (45.6%)	20 (50%)	6 (35.3%)
Residual or recurrent	31 (54.4%)	20 (50%)	11 (64.7%)
Primary tumor location			
Ear	21 (36.8%)	15 (37.5%)	6 (35.3%)
Scalp	15 (26.3%)	9 (22.5%)	6 (35.3%)
Nose	5 (8.8%)	4 (10.0%)	1 (5.9%)
Temporal	3 (5.3%)	2 (5.0%)	1 (5.9%)
Cheek	3 (5.3%)	3 (7.5%)	-
Peri-orbital	3 (5.3%)	1 (2.5%)	2 (11.8%)
Neck	3 (5.3%)	2 (5.0%)	1 (5.9%)
Peri-oral	2 (3.5%)	2 (5.0%)	-
Frontal	2 (3.5%)	2 (5.0%)	-
Stage			
Stage I–II	28 (49.1%)	20 (50.0%)	8 (47.1%)
Stage III–IV	29 (50.9%)	20 (50.0%)	9 (52.9%)
Tumor size, median (range), cm	2.0 (0.2–12.0)	2.0 (0.2–12.0)	2.0 (1.0–6.5)
Tumor type			
Squamous cell carcinoma	36 (63.2%)	24 (60.0%)	12 (70.6%)
Basal cell carcinoma	6 (10.5%)	3 (7.5%)	3 (17.6%)
Malignant melanoma	7 (12.3%)	5 (15.5%)	2 (11.8%)
Merkel cell carcinoma	4 (7.0%)	4 (10.0%)	-
Other *	4 (7.0%)	4 (10.0%)	-
**Treatment characteristics**			
Treatment intensity **			
Minor	21 (38.9%0	13 (33.3%)	8 (53.3%)
Major	33 (61.1%)	26 (66.7%)	7 (46.7%)
Anesthesia			
Local	5 (8.8%)	1 (2.5%)	4 (23.5%)
General	52 (91.2%)	39 (97.5%)	13 (76.5%)
Reconstructive surgery ***			
No	25 (44.6%)	15 (38.5%)	10 (58.8%)
Yes	31 (55.4%)	24 (61.5%)	7 (41.2%)

**Table 2:** Outcomes of the geriatric assessments of patients surgically treated for cutaneous malignancies of the head and neck area. The data are stratified for low SMIs. Due to missingness, not all numbers sum up to 57. ACE-27 = Adult Comorbidity Evaluation 27, ADL = activities of daily living, G8 = Geriatric 8, GDS-15 = Geriatric Depression Scale 15, GFI = Groningen Frailty Indicator, IADL = instrumental activities of daily living, MMSE = Mini-Mental State Examination, MUST = Malnutrition Universal Screening Tool, ND = not determined, TUG = Timed Up and Go.

	Total *n =* 57	Normal SMI *n =* 40 (70.2%)	Low SMI *n =* 17 (29.8%)
**Frailty indicators**			
G8			
Non-frail (>14)	25 (58.1%)	21 (67.7%)	4 (33.3%)
Frail (≤14)	18 (41.9%)	10 (32.3%)	8 (66.7%)
GFI			
Non-frail (<4)	32 (80.0%)	23 (79.3%)	9 (81.8%)
Frail (≥4)	8 (20.0%)	6 (20.7%)	2 (18.2%)
**Comorbidities**			
ACE-27			
None or mild	14 (24.6%)	11 (27.5%)	3 (17.6%)
Moderate or severe	43 (75.4%)	29 (72.5%)	14 (82.4%)
**Polypharmacy**			
Medication count			
< 5 medications	29 (67.4%)	19 (61.3%)	10 (83.3%)
≥5 medications	14 (32.6%)	12 (38.7%)	2 (16.7%)
**Nutritional status**			
MUST			
Low risk	48 (84.2%)	37 (92.5%)	11 (64.7%)
Medium to high risk	9 (15.8%)	3 (7.5%)	6 (35.3%)
**Functional status**			
ADL			
Independent (<2)	53 (93.0%)	37 (92.5%)	16 (94.1%)
Moderate independent (2–4)	4 (7.0%)	3 (7.5%)	1 (5.9%)
IADL			
No restrictions (<1)	16 (37.2%)	10 (32.3%)	6 (50.0%)
Restrictions (≥1)	27 (62.8%)	21 (67.7%)	6 (50.0%)
TUG			
No restrictions (<20)	40 (95.2%)	30 (96.8%)	10 (90.9%)
Restrictions (≥20)	2 (4.8%)	1 (3.2%)	1 (9.1%)
History of falls			
No	46 (90.2%)	32 (88.9%)	14 (93.3%)
Yes	5 (9.8%)	4 (11.1%)	1 (6.7%)
**Social support**			
Education			
Low level	17 (37.0%)	13 (40.6%)	4 (28.6%)
Middle and high level	22 (56.4%)	15 (53.6%)	7 (63.6%)
Relationship			
No	15 (30.6%)	12 (34.3%)	3 (21.4%)
Yes	34 (69.4%)	23 (65.7%)	11 (78.6%)
**Cognitive status**			
MMSE			
Normal cognition (>24)	35 (81.4%)	26 (83.9%)	9 (75.0%)
Declined cognition (≤24)	8 (18.6%)	5 (16.1%)	3 (25.0%)
Risk of delirium			
No	47 (82.5%)	32 (80.0%)	15 (88.2%)
Yes	10 (17.5%)	8 (20.0%)	2 (11.8%)
**Psychological status**			
GDS-15			
No depression (<6)	40 (95.2%)	29 (96.7%)	11 (91.7%)
Depression (≥6)	2 (4.8%)	1 (3.3%)	1 (8.3%)

**Table 3:** Postoperative outcomes for patients surgically treated for skin cancer of the head and neck region. The data are stratified for low SMIs. SMI = skeletal muscle index.

	Total *n =* 57	Normal SMI *n =* 40 (70.2%)	Low SMI *n =* 17 (29.8%)
**Complications**			
No complications	22 (38.6%)	17 (42.5%)	5 (29.4%)
Grade I	9 (15.8%)	6 (15.0%)	3 (17.6%)
Grade II	15 (26.3%)	10 (25.0%)	5 (29.4%)
Grade III	9 (15.8%)	6 (15.0%)	3 (17.6%)
Grade IV	2 (3.5%)	1 (2.5%)	1 (5.9%)
Grade >II (endpoint)	35 (61.4%)	23 (57.5%)	12 (70.6%)
**Hospitalization**			
Duration			
Median (range), days	4.0 (1.0–29.0)	4.0 (1.0–29.0)	3.0 (1.0–22.0)
Missing	1		
Unplanned readmission			
No	51 (89.5%)	36 (90.0%)	15 (88.2%)
Yes	6 (10.5%)	4 (10.0%)	2 (11.8%)

**Table 4:** Univariate linear regression analysis with SMI as the dependent variable and two univariate logistic regression analyses with low SMIs and POC as the dependent variables. Significant *p*-values (α < 0.05) are curved and bold. * indicates that one value was manually added into a blank cell to generate the odds ratios. 95% CI = 95% confidence interval, ACE-27 = Adult Comorbidity Evaluation 27, B = beta, ADL = activities of daily living, BMI = body mass index, G8 = Geriatric 8, GDS-15 = Geriatric Depression Scale 15, GFI = Groningen Frailty Indicator, MUST = Malnutrition Universal Screening Tool, OR = odds ratio, POC = postoperative complication, SD = standard deviation, SMI = skeletal muscle index, TUG = Timed Up and Go.

	SMI		Low SMI		POC	
	B (95% CI)	*p*-Value	OR (95% CI)	*p*-Value	OR (95% CI)	*p*-Value
Age, year	−0.12 (−0.32–0.09)	0.255	1.09 (1.00–1.18)	***0.041***	0.99 (0.93–1.05)	0.750
Sex						
Male	Ref		Ref		Ref	
Female	−8.08 (−11.30–−4.86)	***>0.001***	0.20 (0.04–0.10)	***0.049***	0.50 (0.64–6.25)	0.233
BMI, kg/cm^2^	0.74 (0.33–1.14)	***0.001***	0.58 (0.42–0.80)	***0.001***	0.97 (0.85–1.11)	0.645
Smoking status						
Never	Ref		Ref		Ref	
Former or current	4.61 (1.20–8.02)	***0.009***	0.64 (0.17–2.48)	0.520	3.14 (0.90–11.03)	0.074
Alcohol usage						
None or mild	Ref		Ref		Ref	
Heavy (>2 units/day)	7.23 (1.14–13.31)	***0.021***	0.59 (0.06–5.91)	0.654	3.40 (0.35–33.40) *	0.294 *
Stage						
Stage I–II	Ref		Ref		Ref	
Stage III–IV	2.60 (−0.95–6.15)	0.148	1.13 (0.36–3.51)	0.839	1.93 (0.65–5.68)	0.235
Treatment intensity *						
Minor	Ref		Ref		Ref	
Major	2.76 (−0.83–6.34)	0.129	0.44 (0.13–1.47)	0.182	2.42 (0.77–7.65)	0.131
Anesthesia						
Local	Ref		Ref		Ref	
General	4.34 (−1.95–10.62)	0.172	0.08 (0.01–0.81)	***0.033***	8.24 (0.85–79.44) *	0.068 *
Reconstruction						
No	Ref		Ref		Ref	
Yes	−3.46 (−7.04–0.13)	0.058	0.44 (0.14–1.40)	0.163	1.94 (0.65–5.75)	0.233
G8						
Non-frail (>14)	Ref		Ref		Ref	
Frail (≤14)	−2.09 (−5.94–1.76)	0.281	4.20 (1.02–17.32)	***0.047***	5.42 (1.25–23.49)	***0.024***
GFI						
Non-frail (<4)	Ref		Ref		Ref	
Frail (≥4)	2.17 (−3.01–7.34)	0.405	0.85 (0.14–5.03)	0.860	6.18 (0.68–56.15)	0.106
ACE-27						
None or mild	Ref		Ref		Ref	
Moderate or severe	1.65 (−2.53- 5.83)	0.433	1.77 (0.43–7.38)	0.433	1.27 ( 0.37–4.31)	0.706
MUST						
Low risk	Ref		Ref		Ref	
Medium to high risk	−3.40 (−8.27–1.48)	0.168	6.73 (1.44–31.40)	***0.015***	0.75 (0.18–3.16)	0.695
TUG						
No restrictions (<20 s)	Ref		Ref		Ref	
Restrictions (≥20 s)	−4.90 (−14.64–4.84)	0.318	3.00 (0.17–52.53)	0.452	0.60 (0.04–10.32)	0.725
SMI, cm^2^/m^2^	-	-	-	-	1.02 (0.94–1.10)	0.703
Low SMI						
No	-	-	-	-	Ref	
Yes	-	-	-	-	1.77 (0.53–5.99)	0.356

**Table 5:** A multivariable logistic regression analysis with a low SMI as the dependent variable. Significant *p*-values (α < 0.05) are curved and bold. 95% CI = 95% confidence interval, G8 = Geriatric 8, MUST = Malnutrition Universal Screening Tool, OR = odds ratio.

	Low SMI	
	OR (95% CI)	*p*-Value
Anesthesia		
General	Ref	
Local	0.06 (0.00–1.15)	0.062
G8		
Non-frail (>14)	Ref	
Frail (≤14)	7.68 (1.19–49.66)	***0.032***
MUST		
Low risk	Ref	
Medium to high risk	9.55 (1.19–76.94)	***0.034***

## Data Availability

The data that support the findings of this study are available from the corresponding author upon reasonable request.

## Supplementary Materials

The following supporting information can be downloaded at: https://www.mdpi.com/article/10.3390/jcm12103445/s1, Table S1: The geriatric assessment at baseline showing a range of validated instruments on multiple domains with applied cut-off values. Table S2: Remaining variables of the univariate linear regression analysis with SMI as the dependent variable and two univariate logistic regression analyses with low SMIs and POC as the dependent variables. Table S3: Multivariable linear regression analysis with SMI as the dependent variable [41,42,43,44,45,46,47,48,49,50,51].
