# Supplementary material for: Radiologically Defined Sarcopenia as a Biomarker for Frailty and Malnutrition in Head and Neck Skin Cancer Patients
Source: J Clin Med. 2023 May 13;12(10):3445. doi: 10.3390/jcm12103445 (PMC10218972; doi:10.3390/jcm12103445)
Supplement: Supplementary file 1 [file jcm-12-03445-s001.zip › jcm-2374245-supplementary.pdf]

Table S1. The geriatric assessment at baseline, showing a range of validated instruments on multiple domains with applied cut-off values. ACE-27 = Adult Comorbidity Evaluation 27, ADL = Activities of Daily Living, G8 = Geriatric 8, GDS-15 = Geriatric Depression Scale 15, GFI = Groningen Frailty Indicator, MMSE = Mini Mental State Examination, MUST = Malnutrition Universal Screening Tool, TUG = Timed Up and Go.

	Abbreviation	Cut-off value
**Frailty indicators**		
Geriatrics 8(41)	G8	Frail: G8 ≤ 14
Groningen Frailty indicator(42)	GFI	Frail: GFI ≥ 4
**Comorbidities**		
Adult Comorbidity Evaluation-27(43)	ACE-27	Moderate to severe: ACE-27 ≥2
**Polypharmacy**		
Medication count(44)		Polypharmacy: medications ≥ 5
**Nutritional status**		
Malnutrition Universal Screening Tool(45)	MUST	Medium to high risk: MUST ≥1
**Functional status**		
Activities of Daily Living(46)	ADL	Restrictions: ADL ≥2
Instrumental Activities of Daily Living(47)	IADL	Restrictions: IADL ≥ 1
Timed Up and Go(48)	TUG	Restrictions: TUG ≥ 20
History of falls		Yes
**Social support**		
Education		Low level
Relationship		Not in a relationship
**Cognitive status**		
Mini Mental State Examination(49)	MMSE	Declined cognition: MMSE ≤ 24
Risk of delirium(50)		Yes
**Psychological status**		
Geriatric Depression Scale-15(51)	GDS-15	Depression: GDS-15 ≥ 6

Table S2. Remaining variables of the univariate linear regression analysis with SMI as dependent and the univariate logistic regression analysis with low SMI and POC as dependent**.** Significant P-values (α <0.05) are curved and bold. (*) Manually added one value into blank cell to generate odds ratios. *95%CI = 95% Confidence Interval, ADL = Activities of Daily Living, B = Beta, GDS-15 = Geriatric Depression Scale 15, IADL = Instrumental Activities of Daily Living, MMSE = Mini Mental State Examination, OR = Odds Ratio, POC = Postoperative Complication, SD= standard deviation, SMI = Skeletal Muscle Index.*

	**SMI**		**Low SMI**		**POC**	
	**OR (95% CI)**	**p-value**	**B (95% CI)**	**p-value**	**OR (95% CI)**	**p-value**
Reason for referral						
Primary	Ref		Ref		Ref	
Residual or recurrent	-2.41 (-5.98 – 1.16)	0.182	1.83 (0.57 – 5.92)	0.311	2.44 (0.82 – 7.29)	0.109
Tumor size, cm	0.02 (-0.09 – 0.12)	0.733	1.01 (0.98 – 1.04)	0.684	1.00 (0.97 – 1.03)	0.885
Histopathology						
Keratinocyte	Ref		Ref		Ref	
Other	4.78 (-2.18 – 11.75)	0.172	0.28 (0.06 – 1.40)	0.120	0.64 (0.19 – 2.10)	0.456
Invasion depth , mm	0.05 (-0.83 – 0.92)	0.915	1.12 (0.86 – 1.45)	0.390	1.29 (0.96 – 1.75)	0.094
Medication count						
<5 medications	Ref		Ref		Ref	
≥ 5 medications	3.59 (-0.50 – 7.69)	0.084	0.32 (0.059– 1.70)	0.180	1.77 (0.45 – 6.98)	0.418
ADL						
No restrictions (<2)	Ref		Ref		Ref	
Restrictions (≥2)	-2.68 (-9.73 – 4.36)	0.449	1.30 (0.13 – 13.44)	0.827	2.13 (0.21 – 21.84)*	0.525*
IADL						
No restrictions (<1)	Ref		Ref		Ref	
Restrictions (≥ 1)	-0.36 (-4.39 – 3.66)	0.857	2.10 (0.54 – 8.18)	0.285	0.98 (0.27 – 3.52)	0.976
History of falls						
No	Ref		Ref		Ref	
Yes	-0.96 (-7.35 – 5.43)	0.765	0.57 (0.058 – 5.58)	0.630	0.88 (0.13 – 5.80)	0.894
Education						
Low level	Ref		Ref		Ref	
Middle and high level	2.69 (-0.97 – 6.32)	0.147	1.52 (0.36 – 6.37)	0.570	1.23 (0.33 – 4.49)	0.759
Relationship						
No	Ref		Ref		Ref	
Yes	-0.96 (-7.35 – 5.43)	0.765	1.36 (0.42 – 4.39)	0.612	0.46 (0.12 – 1.74)	0.253
MMSE						

Normal cognition (> 24)	Ref		Ref		Ref	
Declined cognition (≤ 24)	-0.23 (-5.44 – 4.97)	0.929	1.73 – 0.34 – 8.76)	0.506	1.0 (0.20 – 4.82)	0.985
Risk of delirium						
No	Ref		Ref		Ref	
Yes	1.45 (-3.29 – 6.19)	0.542	0.53 (0.10 – 2.82)	0.460	1.6 (0.4 – 6.9)	0.541
GDS-15						
No depression (< 6)	Ref		Ref		Ref	
Depression (≥ 6)	0.30 (-9.53 – 10.13)	0.952	2.64 (0.15 – 45.91)	0.506	2.13 (0.21 – 21.84)*	0.525*

Table S3. Multivariable linear regression analysis with SMI as dependent variable. Significant P-values (α <0.05) are curved and underscored.(*) Adjusted for drinking. *95%CI = 95% Confidence Interval, B = Beta, G8 = Geriatric 8, MUST = Malnutrition Universal Screening Tool, OR = Odds Ratio, SMI = Skeletal Muscle Index.*

	**SMI as dependent**<br>**B (95% CI)**	**p-value**
Sex		
Male	Ref	
Female	-7.36 (-10.56 – -4.16)*	***<0.001****
Smoking		
Never	Ref	
Former or current	3.15 (0.17 – 6.34)*	***0.039****
